# The prevalence of permanent pacemaker implantation after open-heart surgeries; eight years of experience in Tehran heart center

**DOI:** 10.1186/s12872-023-03182-2

**Published:** 2023-03-29

**Authors:** Kaveh Oraii Yazdani, Akbar Shafiee, Amirhossein Heidari, Hossein Ahmadi-Tafti, Ahmad Yaminisharif

**Affiliations:** 1 Department of Cardiology, Tehran Heart Center, Cardiovascular Diseases Research Institute, Tehran University of Medical Sciences, Tehran, Iran; 2 Department of Cardiology, School of Medicine, Zahedan University of Medical Sciences, Zahedan, Iran; 3 Cardiac Primary Prevention Research Center, Cardiovascular Diseases Research Institute, Tehran University of Medical Sciences, Tehran, Iran; 4 Faculty of Medicine, Tehran Medical Sciences, Islamic Azad University, Tehran, Iran; 5 Department of Cardiac Surgery, Tehran Heart Center, Cardiovascular Diseases Research Institute, Tehran University of Medical Sciences, Tehran, Iran; 6 Department of Cardiac Electrophysiology, Tehran Heart Center, Cardiovascular Diseases Research Institute, Tehran University of Medical Sciences, Tehran, Iran; 7 Department of Electrophysiology, Tehran Heart Center, North Kargar Ave, Tehran, 1411713138 Iran

**Keywords:** Permanent pacemaker, Valvular surgery, Coronary artery bypass graft

## Abstract

**Background:**

We aimed to evaluate the prevalence of permanent pacemaker implantation (PPI) among open-heart surgery patients.

**Methods:**

We reviewed data from 23 461 patients undergoing open-heart surgeries between 2009 and 2016 in our heart center in Iran. A total of 18 070 patients (77%) had coronary artery bypass grafting (CABG), 3 598 (15.3%) valvular surgeries, and 1 793 (7.6%) congenital repair procedures. Finally, 125 patients who received PPI following open-heart surgeries were enrolled in our study. We defined the demographic and clinical characteristics of all these patients.

**Results:**

PPI was required in 125 (0.53%) patients with an average age of 58 ± 15.3 years. The average hospitalization time after surgery and waiting time for PPI were 19.7 ± 10.2 and 11.4 ± 6.5 days, respectively. Atrial fibrillation was the dominant pre-operative cardiac conduction abnormality (29.6%). Also, the primary indication for PPI was complete heart block in 72 patients (57.6%). Patients in the CABG group were significantly older (*P* = 0.002) and were more likely to be male (*P* = 0.030). The valvular group longer bypass and cross-clamp times and had more left atrial abnormalities. In addition, the congenital defect group was younger and had longer ICU stay times.

**Conclusions:**

Based on our study findings, PPI was required in 0.53% of patients following open-heart surgery due to damage to the cardiac conduction system. The current study paves the way for future investigations to identify possible predictors of PPI in patients undergoing open-heart surgeries.

## Introduction

Conduction defects are common complications following open-heart surgeries, approximately appearing in 10–15% of patients undergoing open-heart surgeries [1–3]. The lowest incidence of such a complication is seen after CABG, while valvular surgery provides the highest prevalence of permanent pacemaker implantation (PPI) [1, 2]. Since the cardiac conduction system is anatomically located near the cardiac valves, the conduction system can be susceptible to damage during valvular heart disease or surgical-related procedures [4, 5]. In accordance with the European Society of Cardiology (ESC) and the European Association for Cardio-Thoracic Surgery (EACTS) guidelines for the management of valvular heart disease, the benefits of valvular surgeries for patients in the early stages of valvular disease should be carefully evaluated with regard to potential complications that could lead to further interventions [6].

There are several indications for PPI after open-heart surgeries, the most prevalent being bradyarrhythmia-related conditions, such as atrioventricular block (AVB) and sinus node dysfunction or atrial fibrillation (AF) with a slow ventricular response [7, 8]. AVB may occur permanently or temporarily. In the case of transient AVB, patients sometimes need a temporary pacemaker until the recovery of native atrioventricular conduction. However, persistent post-operative AVB necessitates treatment with interventional procedures, and if the cause of AVB is irreversible, the patient often requires PPI [3, 9–11]. Over the past five decades, the development of implantable electronic devices has facilitated the management of conduction disturbances after open-heart surgeries, which have been life-saving for many patients worldwide [8, 11].

Besides, the prevalence of PPI has considerably increased in Iran over the past decades [12, 13]. Nevertheless, there are still no precise data on the predictors, benefits, and prevalence of PPI following open-heart surgeries. Therefore, we aimed to evaluate the prevalence of PPI among patients who underwent open-heart surgeries and define the patients’ characteristics.

## Methods

In this study, we reviewed data from 23 461 patients who underwent open-heart surgeries at Tehran Heart Center between 2009 and 2016 and retrieved information from those who required PPI following surgery. Overall, 18 070 patients (77%) had CABG, 3 598 (15.3%) valvular surgeries, and 1 793 (7.6%) congenital repair procedures, respectively. All the surgeries were performed in Tehran Heart Center operating rooms under standard protocols. In detail about surgery information, St. Thomas’ Hospital cardioplegic solutions were administrated during surgery [14]. A dual-chamber permanent pacemaker implantation was performed for complete heart block and sick sinus patients who required PPI after open-heart surgeries, while a single-chamber permanent pacemaker was implanted for patients with AF. The pacing mode for complete heart block, sick sinus, and AF with ventricular response patients was DDD, DDD®, and VVI, respectively. Postoperatively, a temporary epicardial ventricular single-pacing lead was placed in the operating room after the cardiac procedure was completed and before chest closure. Our center’s policy regarding the decision of PPI was left to the discretion of an expert cardiology team consisting of an interventional cardiologist, cardiothoracic surgeon, and anesthesiologist.

The adult Cardiac Surgery Data Bank of Tehran Heart Center keeps records of open-heart surgeries, and the details of this data bank have been published previously [15, 16]. Based on ESC Guidelines on cardiac pacing and resynchronization therapy criteria for conduction system disease, we included patients with a clinical indication for PPI, such as complete heart block, sick sinus syndrome, slow ventricular response, and AVB within the hospitalization period following cardiac surgery [17].

Exclusion criteria were: previous PPI, previous history of an implantable cardioverter defibrillator (ICD) or cardiac resynchronization (CRT) therapy due to tachyarrhythmia or heart failure, or patients with an indication for PPI before open-heart surgery. In addition, the redo surgeries group was excluded from our study due to a different risk and prognosis level.

All the patients gave informed consent upon reassurances that their clinical data would be used anonymously for research purposes. The institutional board and the medical ethics committee of Tthc approved the study protocol (protocol number: THC95-751).

The general characteristics of the patients included demographic features (age and sex), body mass index, blood pressure, cardiovascular risk factors (hypertension, diabetes mellitus, dyslipidemia, and smoking), a history of cardiac diseases, other remarkable comorbidities, as well as electrocardiographic and echocardiographic data. The definitions of these characteristics have been described elsewhere [18]. The type of surgery and surgery-related variables, such as the pump time and the cross-clamp time, were retrieved from the data bank. In addition, values of all the variables were also reported for the study population.

### Center information

Tehran Heart Center has 460 inpatient beds in 21 state-of-the-art wards on 11 floors [19]. There are 11 operating rooms (consisting of one hybrid operation room), six cath labs, five coronary care units (72 beds), seven post-coronary care units (112 beds), two intensive care units (42 beds), three post-intensive care units (36 beds), nine echocardiography rooms, 18 beds in the emergency department, a research department, a pathology department, a cardiac rehabilitation center, a nuclear medicine department, and a radiology department (equipped with 256-slice CT scanners).

Between 2001 and the end of 2017, approximately 1 300 000 patients were referred to outpatient clinics, more than 800 000 patients visited emergency rooms, over 280 000 patients were hospitalized, and at least 55 000 patients underwent open-heart surgeries at Tehran Heart Center. Furthermore, 150 000 coronary angiographies and 35 000 angioplasties were performed, as well as more than 500 000 echocardiographic studies, over 20 000 pacemakers, ICD and CRT device implantations, electrophysiology tests, and ablation procedures. Aside from hybrid surgery, Tehran Heart Center also performs transcatheter aortic valve implantation (TAVI), valve-in-valve (VIV) procedures, endovascular aortic aneurysm repair (EVAR), and thoracic endovascular aortic repair (TEVAR).

### Statistical analysis

Numerical variables were presented as the mean ± the standard deviation, while categorized variables were shown as frequencies and percentages. Continuous variables were compared using the Student *t*-test or the nonparametric Mann–Whitney *U* test whenever the data did not appear to have a normal distribution, and the χ^2^ test or the Fisher exact test was used for categorical variables. A *P* value of less than 0.05 was considered statistically significant. All the statistical analyses were performed using SPSS version 21 (IBM Corp, USA).

## Results

**Fig. 1:**
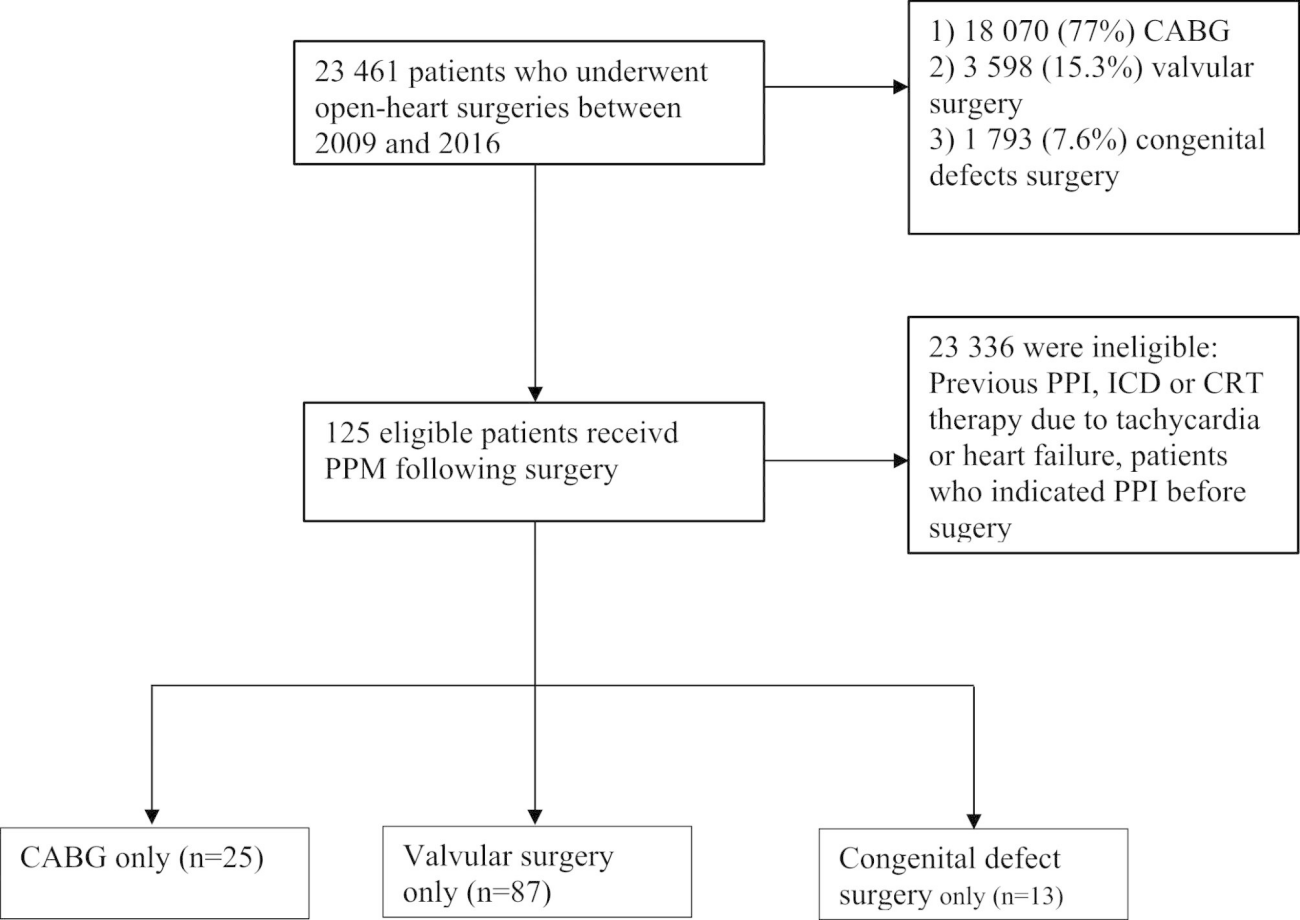
The flowchart represents the inclusion process of our study

In this study, we included 125 eligible patients who met our criteria. A flowchart displays the patient inclusion process in our study (Figure-1). The overall prevalence rate of PPI was 0.53%, and the mean age of the patients was 56.7 ± 15.6 years. Sixty-one patients (48.8%) were male. The prevalence rate of PPI was 0.13%, 2.4%, and 0.72% after CABG, valvular surgery, and congenital defect surgery, respectively. AF was the dominant pre-operative cardiac conduction abnormality (29.6%). Among our study population, ten had left bundle branch block (LBBB), 15 had right bundle branch block (RBBB), 12 had complete heart block, and 10 had First-degree AVB. Besides, among all valvular surgeries, the prevalence rates of PPIs after mitral valve replacement (MVR), aortic valve replacement (AVR), MVR + AVR, MVR + tricuspid valve repair (TVR), and MVR + AVR + TVR were 2.5%, 2.7%, 3.6%, 4.54%, and 15.59%, respectively. The average duration of hospitalization was 19.7 ± 10.2 days. Additionally, the mean time to pacing was 11.4 ± 6.5, and no mortality was reported during the hospitalization period. The details of the general characteristics of the patients, including demographic and clinical characteristics, are illustrated in Table 1.

**Table 1:** Demographic and clinical characteristics of the study population

Characteristics	n = 125
Age, mean ± SD, y	56.7 ± 15.6
Male sex, n (%)	61 (48.8)
BMI > 25, n (%)	64 (51.2)
Diabetes mellitus, n (%)	35 (28.0)
Dyslipidemia, n (%)	40 (32.0)
Hypertension, n (%)	45 (36.0)
Smoking, n (%)	
Current	10 (8.0)
Former	14 (11.2)
Family history of CAD, n (%)	31 (24.8)
Renal failure, n (%)	4 (3.2)
COPD, n (%)	4 (3.2)
CVA, n (%)	8 (6.4)
NYHA class, n (%)	
I, II	82 (65.6)
III	34 (27.2)
Pre-operative ECG abnormalities, n (%)	
AF	37 (29.6)
LBBB	10 (8.0)
RBBB	15 (12.0)
CHB	12 (9.6)
First-degree AVB	10 (8.0)
Type of surgery, n (%)	
CABG	25 (20.0)
Valvular surgery	87 (69.6)
Congenital defect	13 (10.4)
EF < 40%, n (%)	16 (11.9)
Pre-pacing ECG abnormalities	
Slow ventricular response	33 (26.4)
CHB	72 (57.6)
Sick sinus	18 (14.4)
AVB	2 (1.6)
LA size > 40, n (%)	81 (60.0)
Diastolic left ventricular size > 55, n (%)	26 (19.3)
Systolic left ventricular dimension, mean ± SD, mm	35.2 ± 8.4
Duration of hospitalization, mean ± SD, d	19.7 ± 10.2
Cross-clamp time, median [IR], min	70.0 [47.9, 70.0]
Pump time, median [IR], min	112.0 [81.0, 170.0]
ICU stay, mean ± SD, h	125.6 ± 96.8
Time to pacing, mean ± SD, d	11.4 ± 6.5

AF: Atrial fibrillation; AVB: Arterioventricular block; BMI: Body mass index; CABG: Coronary artery bypass graft; CAD: Coronary artery disease; CHB: Complete heart block; COPD: Chronic obstructive pulmonary disease; CVA: Cerebrovascular accident; EF: Ejection fraction; ICU: Intensive care unit; IR: interquartile range; LA: Left atrium; LBBB: Left bundle branch block; NYHA: New York Heart Association; RBBB: Right bundle branch block

The primary indication for PPI was complete heart block in 72 patients (57.6%). Other indications included slow ventricular response in 33 patients (26.4%), sick sinus syndrome in 18 patients (14.4%), and AV block in two patients (1.2%).

In subgroups analysis, patients in the CABG group (66.5 ± 9.4) were significantly older than those in the valvular and congenital defect surgery group (56.4 ± 14.7 and 40.4 ± 17.7, respectively [P < 0.001]). The valvular surgery group was more likely to be male than other groups. In addition, the congenital defect surgery group tended to be younger, with longer ICU stay times (135.3 ± 100.5 vs. 130.7 ± 49.6 valvular surgery group and 123.1 ± 90.5 in the CABG group). The prevalence of chronic comorbidities, including diabetes mellitus, dyslipidemia, hypertension, and smoking, was higher in the CABG group. Additionally, the prevalence of first-degree AVB was notably higher in the CABG group, while AF was observed more frequently among patients in the valvular surgery group. Complete heart block as the primary indication for PPI was more prevalent among all surgery groups. Nearly all patients with slow ventricular response were in the valvular surgery group. AVB block patients were all observed in the CABG group as well. The detailed comparisons among the three different surgery groups are presented in Table 2.

**Table 2:** Comparison of the demographic and clinical characteristics of the CABG, valvular surgery, and congenital defects repair surgery groups

Characteristics	CABG only (n = 25)	Valvular surgery only (n = 87)	Congenital defects surgery only (n = 13)	*P* value*
Age, mean ± SD, y	66.5 ± 9.4	56.4 ± 14.7	40.4 ± 17.7	< 0.001
Male sex, n (%)	17 (68.0)	38 (43.6)	6 (46.2)	0.098
BMI > 25, n (%)	13 (52.0)	46 (52.9)	5 (38.5)	0.622
Diabetes mellitus, n (%)	13 (52.0)	21 (24.1)	1 (7.7)	0.005
Dyslipidemia, n (%)	15 (60.0)	22 (25.3)	3 (23.1)	0.004
Hypertension, n (%)	14 (56.0)	28 (32.2)	3 (23.1)	0.054
Smoking, n (%)				0.344
Current	3 (12.0)	7 (8.0)	0 (0)	
Former	5 (20.0)	8 (9.2)	1 (7.7)	
Renal failure, n (%)	3 (12.0)	1 (1.1)	0 (0)	0.02
COPD, n (%)	1 (4.0)	3 (3.4)	0 (0)	0.779
	3 (12.0)	5 (5.7)	0 (0)	0.323
NYHA class, n (%)				0.3
I, II	18 (72.0)	53 (60.9)	10 (76.9)	
III, IV	6 (24.0)	28 (32.2)	1 (7.7)	
Pre-operative ECG abnormalities, n (%)				< 0.001
AF	0 (0)	36 (42.3)	1 (7.7)	
RBBB	4 (16.0)	8 (9.2)	3 (23.1)	
LBBB	0 (0)	9 (10.3)	1 (7.7)	
CHB	6 (24)	6 (6.9)	0 (0)	
First-degree AVB	6 (24.0)	2 (2.3)	2 (15.4)	
EF < 40, n (%)	3 (12.0)	12 (13.8)	0 (0)	0.361
Pre-pacing ECG abnormalities, n (%)				0.001
Slow ventricular response	0 (0)	32 (36.8)	1 (7.7)	
CHB	19 (76.0)	43 (49.4)	10 (76.9)	
Sick sinus	4 (16.0)	12 (13.8)	2 (15.4)	
AVB	2 (8.0)	0 (0)	0 (0)	
Duration of hospitalization, mean ± SD, d	14.5 ± 5.0	21.2 ± 10.9	20.3 ± 9.8	0.015
Cross-clamp time, median [IR], min	56.0 [39.0, 78.5]	93.0 [62.0, 120.0]	65.0 [40.7, 99.7]	0.002
Pump time, median [IR], min	89.7 [58.2, 131.0]	109.0 [97.0, 210.0]	96.0 [82.5, 158.0]	0.012
ICU stay, mean ± SD, h	123.1 ± 90.5	130.7 ± 49.6	135.3 ± 100.5	0.934
Time to pacing, mean ± SD, d	9.2 ± 4.1	11.7 ± 6.6	12.8 ± 6.1	0.138

AF: Atrial fibrillation; AVB: Atrioventricular block; BMI: Body mass index; CABG: Coronary artery bypass graft; CHB: Complete heart block; COPD: Chronic obstructive pulmonary disease; CVA: Cerebrovascular accidend; EF: Ejection fraction; ICU: Intensive care unit; IR: interquartile range; NYHA: New York Heart Association; LBBB: Left bundle branch block; RBBB: Right bundle branch block

* P < 0.05 was considered significant.

## Discussion

This study reported patients’ demographic, clinical, and procedural characteristics requiring PPI following open-heart surgeries. Among all our patients after open-heart surgery, we found that the prevalence of PPI was 0.53%, which is significantly lower than in other similar studies [3, 11, 20, 21]. Following CABG, the rate of post-operative PPI was 0.13%, while figures of 0.73% [22], 0.9%[23], 1% [3], and 1.3%[24] were reported in other studies. The prevalence of PPI in the AVR subgroup was 2.7%, whereas related investigations illustrated prevalences of 5.7% [3], 6% [25], 6% [23], 6.2% [22], 6.6% [26], and 9.3% [27] which were substantially higher than our result. Continuing with the 2.5% in the MVR group of our study, 1.8% [3], 4.5% [27], 7.6% [23], and 8.2% were disclosed by other related evidence. PPI prevalence in the congenital heart surgery group was 0.72% compared to a prevalence rate of 1% [28, 29] in recent studies. Moreover, the expected time between surgery and PPI was 11 days, which is in line with previous studies [1, 3, 21, 23]. Besides, we recruited considerably more patients who were younger as well.

Our study had a shorter pump time and cross-clamp time (132, 84 min) in comparison with Huynh et al. [30] (157, 125 min), Merin et al. [3] (141, 92 min), and Raza et al. [23] (168, 121 min) studies. Interstingly, PPI postoperatively is associated with prolonged pump and cross-clamp time [11]. Merin et al. revealed that prolonged cross-clamp time could significantly increase the risk of PPI (*P* < 0.001). Furthermore, Baerman et al. proposed that pump and cross-clamp times were correlated with increased AV conduction defects leading to permanent pacing [31]. These findings may explain the low frequency of PPI in our study population.

Consistent with prior reports, it has long been recognized that post-operative conduction disturbances that require PPI are common complications following valvular surgery [5, 20, 27]. Our result disclosed that multivalvular surgeries had a significantly greater risk of PPI, especially in subgroups of AVR + MVR + TVR, compared to isolated valve procedures. In line with this result, PPI was also more prevalent among patients who had undergone MVR + TVR and MVR + AVR than isolated valve surgery [8, 10, 27, 32]. Moreover, previous literature described that MVR was associated with the highest risk of PPI among isolated valve procedures [33]. In contrast, our results revealed that TVR was associated with the highest risk of PPI among patients who underwent single-valve surgery.

The presence of conduction disturbances at baseline was strongly associated with increased PPI risks during hospitalization [21, 34, 35]. For instance, AF has been shown to be the dominant pre-operative cardiac conduction abnormality associated with a higher requirement of PPI [1, 22, 34, 36]. In addition, previous investigations proposed that conduction system blocks as indications for PPI were the most common conduction disturbances following open-heart surgery [1, 8, 37]. Owing to this, we found that complete heart block was the most common indication for PPI in our study population.

Multiple reports suggest that older age and the presence of chronic comorbidities, including cardiovascular disease, diabetes mellitus, and chronic kidney disease, were observed to be associated with a higher risk of PPI [27, 38, 39]. These predisposing comorbidities with several mechanisms can cause advanced conduction disturbances, such as systemic inflammation, ischaemic injury, remodeling, and cardiac tissue fibrosis. In our study, a high proportion of patients requiring PPI were old and had a history of renal failure, dyslipidemia, and diabetes mellitus.

Eventually, it is necessary to manage eligible patients accurately, detect risk factors, reduce surgery time, and use less invasive procedures to reduce the number of patients requiring PPI after open-heart surgery.

### Study limitations

This study has several limitations. First, this was a retrospective study, and it was impossible to include all the variables related to this topic. Second, this study lacks a control group of matched patients; therefore, we could not identify the predictors for PPI following cardiac surgery. We had no tricuspid valve replacement (all cases were repaired). As for the mitral valve, we considered MV repair and MV replacement in one group.

## Conclusions

As a result, we reported a PPI prevalence of 0.53% at Tehran Heart Center, which is relatively low compared to similar studies. In the subgroup analysis, PPI prevalence was 0.13%, 2.4%, and 0.72% in CABG, valvular, and congenital defect surgery groups, respectively. Identifying the prevalence of adverse open-heart surgery outcomes that necessitate PPI can help clinicians better inform patients about the prognosis of the surgery. In addition, it provides cardiologists with an overview of what to expect regarding conduction defects after surgeries. The current study paves the way for future investigations to identify possible predictors of PPI in patients undergoing open-heart surgeries.

## Data Availability

The data underlying this article will be shared on a reasonable request to the corresponding author.
